# Recent advances in preventing stroke recurrence

**DOI:** 10.12688/f1000research.11597.1

**Published:** 2017-06-28

**Authors:** J David Spence

**Affiliations:** 1 Robarts Research Institute, Western University, London, ON, Canada

**Keywords:** stroke recurrence, hypertension, diet, B12 deficiency, anticoagulation, antiplatelet, intracranial stenosis, LDL cholesterol

## Abstract

Recent advances in secondary stroke prevention include new evidence in hypertension, nutrition, anticoagulation, antiplatelet therapy, intracranial stenosis, percutaneous closure of patent foramen ovale, and lipid-lowering therapy. Individualized therapy for hypertension based on phenotyping with plasma renin and aldosterone markedly improves blood pressure control in patients with resistant hypertension. A Mediterranean diet can reduce the risk of stroke by nearly half. The diagnosis and treatment of metabolic vitamin B12 deficiency, and B vitamins to lower homocysteine, can reduce the risk of stroke by approximately 30%. There are problems with clopidogrel that can be overcome by using ticagrelor, and new anticoagulant drugs markedly improve anticoagulation for stroke prevention, particularly in atrial fibrillation. There are pharmacokinetic problems with dabigatran that deserve attention. Intensive medical therapy is better than stenting for intracranial stenosis, and new therapies directed at proprotein convertase subtilisin–kexin type 9 (PCSK9) will revolutionize lipid-lowering therapy. In the past, it was estimated that rational therapy could reduce recurrent stroke by about 80%. With recent advances, we should be able to do even better.

## Introduction

In 2013, I reviewed intensive medical therapy for secondary stroke prevention ^1^. Since then, there have been a number of important advances, which are reviewed in this update. These include advances in hypertension, nutrition, anticoagulation, antiplatelet therapy, intracranial stenosis, percutaneous closure of patent foramen ovale, and lipid-lowering therapy.

## Hypertension

A major preventable cause of stroke is hypertension, which rose to the top of the list of causes of death and disability in the world in 2012 ^2^. It is a particular problem in Africa and among African-Americans. In Sub-Saharan Africa and in the US, strokes among Africans and African-Americans are disproportionately due to hypertensive small vessel disease (hypertensive lacunar infarctions and intracranial hemorrhage). African-American adults have among the highest prevalence of hypertension in the world: 45% in men and 46% in women ^3^. They also have the highest risk of hemorrhagic stroke ^4^. African-Americans are more likely to have their hypertension diagnosed, treated, and treated more intensively but less likely to have it controlled ^5^.

Salt and water retention may have a survival advantage in conditions of heat and salt and water privation in sub-Saharan Africa ^6^. Grim *et al*. ^7, 8^ hypothesized that a further advantage of salt and water retention was conferred on slaves who survived the Atlantic crossing between decks on slave ships and aided their survival for the first few years of working on plantations in the Southern US. Reasons for the excess of hypertension and poorer control of hypertension in blacks may have some of their origin in socioeconomic conditions but also clearly are based on genetic factors causing salt and water retention ^6^. These are of two types: an increase in primary aldosteronism due to bilateral adrenocortical hyperplasia, and mutations affecting the retention of salt and water via the renal tubular epithelial sodium channel (ENac) ^9^. The former includes variants of *CYP11B2* (aldosterone synthase); the latter includes mutations of ENac itself ( *SCNN1B*) and mutations of other genes affecting the function of ENac ^10^, including *NEDD4L*, *GRK4*, *UMOD*, *NPPA*, and *CYP4A11* ^11^. Among patients with uncontrolled hypertension in Africa, many variants of these genes were present, and some of the variants were present in all participants studied ^10^.

The potential for the reduction of hemorrhagic stroke by the control of hypertension was illustrated in a community-based experience in London, Canada, between 1978 and 1983 and in the North American Symptomatic Carotid Endarterectomy Trial (NASCET). A major push to improve blood pressure control initiated by the Department of Family Medicine in London ^12^, Canada, between 1978 and 1983 resulted in the detection of hypertension in 94% of the community, treatment in 92%, and control in 72% ^13^. This was aided by the initiation in 1977 of a hypertension clinic basing the management of resistant hypertension on levels of stimulated plasma renin activity. By 1983, strokes in London were reduced by half; the strokes that were prevented were entirely those due to hypertensive small vessel disease ^14^. At Victoria Hospital in London, Canada, the number of patients per year with hypertensive intracerebral hemorrhage declined from 200/year to almost none.

In the NASCET trial ^15^, investigators routinely received a stiff letter reminding them to follow the protocol whenever a participant’s blood pressure at a follow-up visit was above the specified target (140/90) and antihypertensive medication was not increased. In the medical arm of the study, this approach virtually eliminated therapeutic inertia and resulted in virtual elimination of hemorrhagic strokes: they were reduced to 0.5% of strokes at a time when approximately 20% of strokes in North America were hemorrhagic, and this included lobar strokes and subarachnoid strokes, which are not due to hypertensive small vessel disease; therefore, therapeutic inertia can be overcome. A remaining important cause of uncontrolled hypertension is “diagnostic inertia”—failure to ask what the underlying cause of the hypertension is when blood pressure is not controlled with usual therapy ^16, 17^.

In 2006, I hypothesized ^18^ that much of the stroke disparity among African-Americans could be eliminated by physiologically individualized therapy (PhysRx) based on phenotyping by plasma renin and aldosterone. That hypothesis has now been supported by a clinical trial in Africa.

Patients with uncontrolled hypertension attending clinics in Nigeria, Kenya, and South Africa were allocated to usual care based on the guidelines in place in each country versus physiologically individualized therapy based on phenotyping by plasma renin and aldosterone ^19^. The study was based on observations among descendants of escaped slaves who made their way to Canada via the Underground Railroad to North Buxton, Ontario, one of four such settlements established in Canada in the 19th century ^20^. The algorithm used to guide therapy is shown in Table 1. Patients with low renin/high aldosterone (primary aldosteronism phenotype) are best treated with aldosterone antagonists, those with low renin/low aldosterone (Liddle phenotype) are best treated with amiloride (a specific antagonist of ENac), and those with high renin/high aldosterone (secondary hyperaldosteronism, a renal phenotype) are best treated with angiotensin receptor blockers (ARBs).

**Table 1.** Physiologically individualized therapy * based on renin/aldosterone profile.

	Primary hyper-aldosteronism	Liddle’s syndrome and variants (renal Na ^+^ channel mutations)	Renal/renovascular
Renin	Low **	Low	High
Aldosterone	High **	Low	High
Primary treatment	Aldosterone antagonist (spironolactone or eplerenone) Amiloride for men where eplerenone is not available (rarely surgery)	Amiloride	Angiotensin receptor blocker or renin inhibitor *** (rarely revascularization)

*It should be stressed that this approach is suitable for tailoring medical therapy in resistant hypertensives; further investigation would be required to justify adrenalectomy or renal revascularization

**Levels of plasma renin and aldosterone must be interpreted in the light of the medication the patient is taking at the time of sampling. In a patient taking an angiotensin receptor blocker (which would elevate renin and lower aldosterone), a plasma renin level that is in the low normal range for that laboratory with a plasma aldosterone level in the high normal range probably represents primary hyperaldosteronism for the purposes of adjusting medical therapy.

***Angiotensin converting enzyme (ACE) inhibitors are less effective because of aldosterone escape via non-ACE pathways such as chymase and cathepsin.

Reproduced by permission of Elsevier from 9.

In the study, there was no effect of the algorithm at the site in Kenya, where low socioeconomic status and perhaps cultural factors limiting adherence resulted in no difference between usual care (UC) and PhysRx. The results of the study were as follows ^19^: “in the overall study population, control of both systolic and diastolic pressures was obtained in 11.1% of UC vs. 50.0% of PhysRx (p = 0.0001). Systolic control was achieved in 14% of UC vs. 60% of PhysRx ( *P* = 0.0001); diastolic control in 36% of UC vs. 67% of PhysRx ( *P* = 0.003). Control was significantly worse in Kenya. When only the sites in Nigeria and South Africa were considered, systolic control was obtained in 15% of UC vs. 79% of PhysRx ( *P* < 0.0001), diastolic control in 45% vs. 71% ( *P* = 0.04), and control of both in 15% vs. 67% ( *P* = 0.0001). If only the Nigerian site (where patients were randomized to the two treatment strategies, *and conditions were more similar to those in North America*) is considered, systolic control was obtained in 15% of UC vs. 85% of PhysRx ( *P* = 0.0001), diastolic control in 45% vs. 75% ( *P* = 0.11), and control of both systolic and diastolic pressure in 15% vs. 75% ( *P* < 0.0001) even though the renal function was worse at that site”. Thus, it is clear that if diagnostic inertia can be overcome, there is a marked improvement in blood pressure control. The cost of the two blood tests (around $50) is very low compared with the cost of high doses of the wrong medication and the cost of events such as stroke, renal failure, and heart failure from uncontrolled hypertension. This approach should be tested in a randomized trial in the US.

## Nutrition

### Mediterranean diet

Diet is far more important than most physicians (and the public) suppose. In the Lyon Diet Heart Study ^21^, there was a greater than 60% reduction of stroke and myocardial infarction over 4 years in secondary prevention. This was approximately twice the effect of simvastatin in the contemporaneous Scandinavian Simvastatin Survival Study ^22^ (a 40% reduction of recurrent myocardial infarction in 6 years). In the US, the worst of the lifestyle and risk factor issues is diet: only 0.1% of Americans consume a healthy diet, and only 8.3% consume even a moderately healthy diet ^3^.

Adherence to a Mediterranean diet reduces the risk of ischemic stroke ^23^, adherence to a healthy diet and other lifestyle factors reduced myocardial infarction in Swedish men by 80% ^24^, and a healthy diet and other lifestyle factors reduced stroke in Swedish women by 62% ^25^. In 2013, it was already clear that, compared to a low-fat diet, a Mediterranean diet significantly reduced stroke in primary prevention: there was a 47% reduction of stroke in the Mediterranean arm of the study fortified with mixed nuts ^26^. Since then, it has been reported that the Mediterranean diet improved the metabolic syndrome ^27^ and reduced age-related cognitive decline ^28^.

### B vitamin therapy to lower homocysteine

In 2004, the Vitamin Intervention in Stroke (VISP) trial ^29^ reported no benefit of folic acid 2.5 mg, pyridoxine 25mg, and cyanocobalamin 400 μg daily compared with low-dose vitamins. In 2006, when the Norwegian Vitamin Trial (NORVIT) ^30^ and the Heart Outcomes Prevention Evaluation (HOPE-2) trial ^31^ were published, the cant was “homocysteine is dead”. Loscalzo hypothesized ^32^ that harm from unmetabolized folic acid may have accounted for the null results. However, in NORVIT, there was harm from B vitamins in the arm of the study that included cyanocobalamin, and in HOPE-2 there was actually a 23% reduction of stroke with B vitamins. In the French SU.FOL.OM3 trial ^33^, there was also a 43% reduction of stroke in a study in which renal function was better than in earlier studies: mean serum creatinine in SU.FOL.OM3 was 78 mmol/L compared with 99.9 mmol/L in VISP, 91 mmol/L in NORVIT, and 88.4 mmol/L in HOPE-2. Some meta-analyses also indicated a reduction of stroke with B vitamins ^34, 35^.

Importantly, the form of vitamin B12 used in all of these studies was cyanocobalamin, a form that contains cyanide, which must be decyanated to become active. Cyanide is converted to thiocyanate before renal elimination occurs, consuming hydrogen sulfide (H _2_S), a gasotransmitter ^36^ that is an endothelium-derived relaxing factor, in the process, and thiocyanate is a powerful oxidant, accelerating the oxidation of LDL cholesterol (LDL-C) ^37^. It accumulates in renal failure ^38^. In the Western Norwegian study (WENBIT) ^39^, cyanocobalamin lowered total homocysteine (tHcy) but not asymmetric dimethylarginine (ADMA), a nitric oxide antagonist that is elevated in the presence of elevated levels of tHcy. In 2010, Koyama found that in patients with renal failure, methylcobalamin lowered both tHcy and ADMA ^40^.

**Figure 1.**
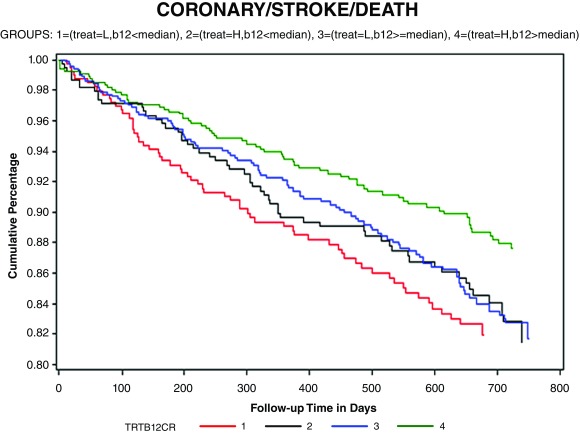
Kaplan-Meier survival free of stroke/myocardial infarction/vascular death in the Vitamin Intervention for Stroke Prevention (VISP) trial excluding patients who received vitamin B12 injections outside the randomized therapy and those with impaired renal function (estimated glomerular filtration rate <46 mL/minute/1.73 m²). Participants were stratified by the median baseline serum B12 level (to characterize the adequacy of B12 absorption) and by randomized allocation to low-dose B vitamins versus high-dose B vitamins (B6 25 mg, B12 400 μg, and folic acid 2.5 mg versus B6 200 μg, B12 6 μg, and folic acid 20 μg daily). The red line represents low-dose vitamins/B12 <median, the green line is high-dose vitamins/B12 above the median, the blue line is low-dose vitamins/B12 above the median, and the black line is high-dose B vitamins/B12 below the median. There was a 34% reduction of the composite endpoint of stroke/myocardial infarction/vascular death in the group with adequate B12 absorption receiving high-dose B vitamins (green line) and the group with less adequate B12 absorption receiving low-dose vitamins. The log-rank p value for all four groups was 0.02. CI, confidence interval; HR, hazard ratio. Reproduced by permission of Wolters Kluver from 42.

In 2011, Spence and Stampfer hypothesized ^41^ that it may have been harm from cyanocobalamin among participants with impaired renal function that accounted for the null result in VISP. That was based on two studies: a subgroup analysis of the VISP trial excluding participants with renal impairment and a study in patients with diabetic nephropathy, the Diabetic Intervention with Vitamins in Diabetic Nephropathy (DIVINe) study. The VISP subgroup analysis was undertaken because in VISP there were several factors that mitigated against an effect of B vitamin therapy. The first was that folate fortification of the grain supply in North America coincided with the initiation of the trial, thereby limiting the benefit of folic acid. The second was that participants in the low-dose arm of the study received the recommended daily allowance of vitamin B12. The third was the decision to give injections of cyanocobalamin to all participants with low B12 levels at baseline, in both the high-dose and the low-dose arms of the study, thus negating the benefit of B12 in the very participants who stood to benefit most. In the subgroup analysis ^42^, we excluded participants who received B12 injections and, for the wrong reason, also excluded participants in the lowest 10% of estimated glomerular filtration rate (eGFR), which was <46 mL/minute/1.73 m² (based on an earlier study ^43^, we thought participants with renal failure would not respond as well to B vitamins; we never dreamed they would be harmed by high-dose B vitamins). To assess the adequacy of B12 absorption, the study population was stratified by the median serum B12 of 322 pmol/L. As shown in Figure 1, there was a 34% reduction of stroke/myocardial infarction/vascular death among participants with a serum B12 level above the median receiving high-dose B vitamins versus those with a serum B12 level below the median receiving low-dose B vitamins.

**Figure 2.**
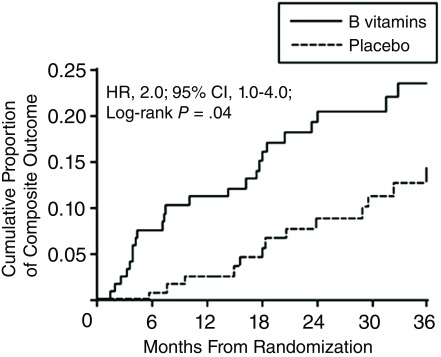
Cumulative proportion of myocardial infarction, stroke, revascularization, and all-cause mortality with high-dose B vitamins in patients with diabetic nephropathy. Diabetic nephropathy patients receiving folic acid 5 mg, B6 25 mg and B12 1,000 μg daily had twice the risk of events compared to patients receiving placebo. All of the events occurred in patients with a glomerular filtration rate of <50 mL/minute/1.73 m² ^45^. Reproduced by permission of the American Medical Association from 44.

Then, in 2010, we found in the DIVINe trial ^44^ that high-dose B vitamins were harmful in patients with impaired renal function. As shown in Figure 2, there was a doubling of stroke, myocardial infarction, and mortality among participants receiving folic acid 5 mg, vitamin B6 25 mg, and vitamin B12 1,000 μg daily compared with those receiving placebo. All of the events occurred among participants with a GFR of <50 mL/minute/1.73 m² ^45^.

The final piece of the puzzle fell into place when the China Stroke Primary Prevention Trial (CSPPT) reported in 2015 ^46^ a significant 25% reduction of stroke with folic acid versus placebo in hypertensive patients receiving the ACE inhibitor enalapril. Among participants with LDL-C above 2 mmol/L, the benefit of folic acid was greater: a 31% reduction in stroke ^47^. Importantly, folic acid was beneficial in participants with impaired renal function ^48^. A meta-analysis stratified by renal function and dose of cyanocobalamin supports the hypothesis that the reason the early trials of B vitamin therapy for stroke prevention were null was due to harm from cyanocobalamin among participants with impaired renal function ^49^, cancelling out benefit among those with good renal function. The implication of all of this is that we should be using methylcobalamin, not cyanocobalamin, for stroke prevention.

### Metabolic B12 deficiency

**Figure 3.**
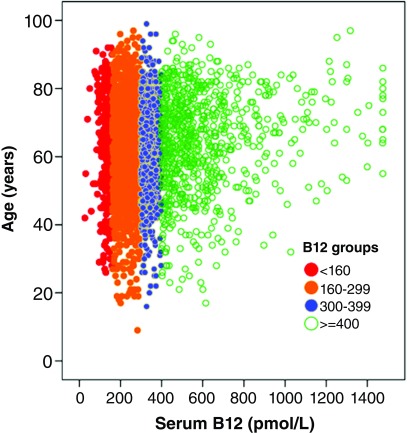
Serum B12 levels among patients attending a stroke prevention clinic. Among 3,025 patients attending a stroke prevention clinic, only 26% had a clearly adequate level of serum B12 >400 pmol/L; of those, many (as seen by the very high levels) were taking B12 supplements. Reproduced by permission of Elsevier from 30.

In 2015, I reviewed the evidence that metabolic B12 deficiency is very common and usually undiagnosed ^50^. It is usually undiagnosed because physicians tend to think that a serum B12 level in the reference range (about 160–600 pmol/L) is adequate; however only a small and variable proportion of total serum B12 is active (about 6–20%). In the low end of the normal range of serum B12, further testing is required to assess the adequacy of functional B12. It is necessary to measure holotranscobalamin, or the metabolites that become elevated in B12 deficiency: methylmalonic acid (MMA), the specific metabolite, or tHcy, which in patients who are folate replete is a reasonable surrogate for MMA. It is difficult to measure MMA, and quantification is not routinely available. Since folate fortification of the grain supply in North America has made folate deficiency rare, tHcy is usually assessed for this purpose ^51^. In the National Health and Nutrition study, the serum B12 level below which tHcy began to rise was approximately 400 pmol/L ^52^; in the Hordaland study, a serum B12 level below 400 pmol/L was also the level below which levels of MMA began to increase ^53^. Thus, to be clearly adequate, the serum B12 level needs to be above 400 pmol/L; as shown in Figure 3, only 26% of patients attending a stroke prevention clinic have such levels of serum B12 ^50^. Among patients attending a stroke prevention clinic, metabolic B12 deficiency was present in 10% of patients below the age of 50 and 30% above the age of 70 ^54^.

**Figure 4.**
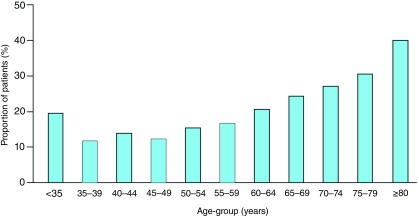
Plasma total homocysteine by age group in vascular patients. Among 2,372 patients referred to our stroke prevention clinic or vascular prevention clinic, the proportion with concentrations of plasma total homocysteine of 14 μmol/L or more increased significantly with age. Reproduced by permission of Elsevier from 58.

Why does this matter? Besides causing neuropathy, myelopathy, and dementia, B12 deficiency elevates levels of tHcy, a clotting factor that is increased in patients with carotid microemboli ^55^ and increased in patients with paradoxical embolism ^56^ and quadruples the risk of stroke in atrial fibrillation ^57^. There is a perfect storm in the aging of the population—the much higher prevalence of atrial fibrillation in the elderly and the very high prevalence of elevated tHcy in the elderly; 40% of vascular patients above the age of 80 had tHcy levels >14μmol/L ^58^ ( Figure 4).

## Anticoagulation

In recent years, oral anticoagulants have become available to replace warfarin. This is a great development because warfarin is impossible to use well. Even in carefully controlled clinical trials, the international normalized ratio (INR) is in the therapeutic range only around 60% of the time. Reasons for this include genetic variation in both the metabolism of warfarin and the susceptibility to a given level of warfarin ^59^ as well as a plethora of interactions with warfarin ^60^. New oral anticoagulants include dabigatran, rivaroxaban, apixaban, and edoxaban (in order of appearance on the market). An important advantage of these drugs is that frequent blood tests are not needed. Unfortunately, all of these drugs are renally excreted, so there are problems with patients with impaired renal function, which includes the elderly ^61^. Among the new oral anticoagulants (NOACs) available, the one with the greatest renal excretion is dabigatran. Furthermore, dabigatran is also the one with the lowest bioavailability (only 6%). This means that drug interactions or a change in absorption would have a much bigger effect on levels of dabigatran than on the other NOACs. There is evidence that dabigatran levels should probably be monitored ^62^, thus eliminating for dabigatran one of the main advantages of NOACs.

## Antiplatelet therapy

An important issue for clopidogrel is that it is an inactive prodrug that requires metabolism via CYP2C19 to the active metabolite. Among Chinese people, 59% are carriers of a loss-of-function allele that results in lack of efficacy of clopidogrel ^63^. Ticagrelor, an antiplatelet agent that acts by the same mechanism (inhibition of the platelet ADP receptor P2Y12), avoids this problem because the parent drug is active ^64^. Although in the main trial ticagrelor did not significantly reduce stroke, it has been shown that ticagrelor reduced stroke among Asian participants ^65^ and also that ticagrelor reduced recurrent stroke among study participants with large artery disease ^66^. As a clinical pharmacologist, I expect that ticagrelor will replace clopidogrel for secondary stroke prevention. It is also likely that dual antiplatelet therapy will be better than monotherapy for high-risk patients; this has not yet been tested with aspirin and ticagrelor.

## Percutaneous closure of patent foramen ovale

There have been important recent advances in percutaneous closure of patent foramen ovale (PFO). This issue has been difficult because 25% of the population have a PFO, but only approximately 5.5% of strokes are due to paradoxical embolism. This means that, in most patients with stroke and a PFO, the PFO is incidental, and there is a problem with statistical power in randomized trials.

Although the early studies of PFO closure did not show significant benefit individually in intent-to-treat analyses, a pooled analysis of individual data from three trials did show significant benefit of closure ^67^. A manuscript is in preparation reporting the benefit of closure in a more recent trial of two new devices. There are occasional serious complications of percutaneous closure, and there is an increased risk of atrial fibrillation following closure, so the selection of patients is important.

There are ways to identify which patients are more likely to benefit from closure: there are clinical clues to paradoxical embolism, and a large shunt on transcranial Doppler (TCD) is predictive of recurrent stroke. Clinical clues include previous pulmonary embolism, deep vein thrombosis or varicose veins, waking up with stroke, a history of migraine, prolonged sitting, a history of sleep apnea, and a Valsalva maneuver at the onset of stroke ^56^. Saline studies with TCD are more sensitive to detect PFO than is trans-esophageal echocardiography (TEE), which missed 15% of right–left shunts (among which over 40% were large shunts) ^68^. Large shunts on TCD were more predictive of recurrent events than was the presence of a shunt or atrial septal mobility on TEE ^68^.

## Intracranial stenosis

Intracranial stenosis is more common in Asian patients and in diabetics ^69, 70^. The Stenting and Aggressive Medical Management for Preventing Recurrent Stroke in Intracranial Stenosis (SAMMPRIS) trial ^71^ reported in 2014 that intensive medical therapy that included lifestyle modification was more efficacious than stenting for intracranial stenosis. In addition to statins and blood pressure treatment, initial dual antiplatelet therapy and intensive lifestyle modification throughout the study were key elements of successful medical therapy.

## Lipid-lowering therapy

Therapy to lower levels of LDL-C clearly reduces the risk of recurrent stroke, particularly in patients with large artery disease ^72^. However, there are important problems with persistence with statins, in part due to adverse effects. After 2 years, less than half of patients prescribed statins are still taking them. The truly causal adverse effects of statins, myopathy and a small increase in the risk of diabetes, are probably due largely to the depletion of ubiquinone (CoQ10) ^73, 74^.

A major recent development in lipid-lowering therapy has been in therapies directed at proprotein convertase subtilisin–kexin type 9 (PCSK9), a protein that results in higher levels of LDL-C by reducing the number of active LDL receptors. Injection of antibodies to PCSK9 and RNA-silencing therapy reduce levels of LDL-C by more than half. Evolocumab, one of the antibody therapies, reduced the composite endpoint of stroke, myocardial infarction, and cardiovascular death by 20% in only 2 years ^75^; lifetime reduction of events would be expected to be much greater. In a *post hoc* analysis, there was a 48% reduction in 78 weeks, of a composite endpoint of death from coronary heart disease, non-fatal myocardial infarction, fatal or nonfatal ischemic stroke, or unstable angina requiring hospitalization with alirocumab ^76^. Tall has suggested ^77^ that the marked reduction of vascular risk observed in persons with loss-of-function mutations of PCSK9 (and therefore lifelong reduction of LDL-C to the same extent as therapy with statins) means that we should be using lipid-lowering therapy earlier in life among persons at risk of vascular disease. As pointed out by Wilkins ^78^, this essentially means all North Americans who aspire to attain old age.

**Figure 5.**
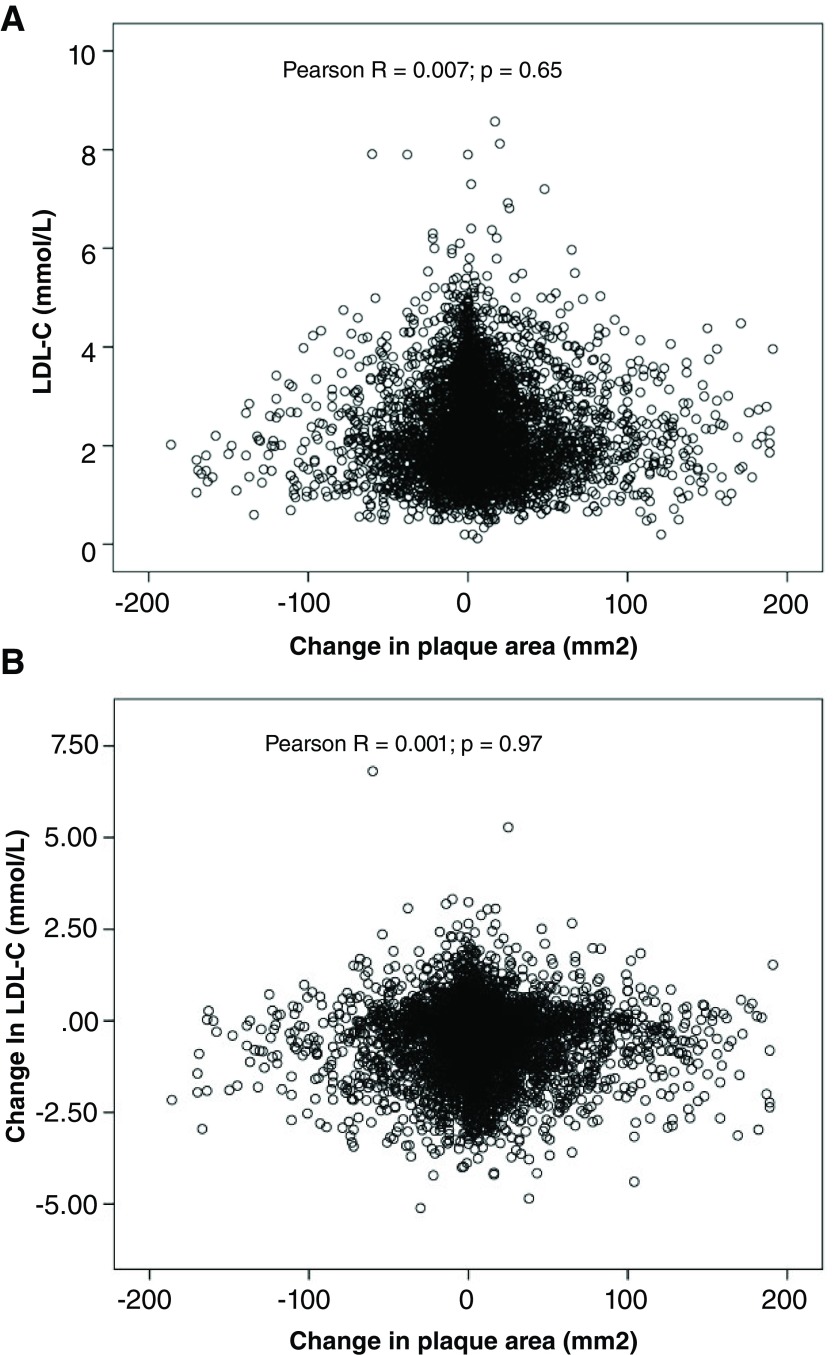
Correlation of low-density lipoprotein cholesterol (LDL-C) (mmol/L), change in LDL-C, and change in total plaque area (mm ^2^). Among 4,512 patients followed in a stroke prevention clinic, there was no correlation between achieved LDL-C with lipid-lowering therapy and change in carotid burden over 1 year (panel **A**). Similarly, there was no correlation between change in LDL-C from baseline to follow-up and change in plaque burden (panel **B**).

In 2003, in our prevention clinics we initiated a strategy of “treating arteries instead of treating risk factors”; the goal of therapy is to stop the progression of atherosclerosis or achieve regression. Among patients with asymptomatic carotid stenosis, this strategy reduced the risk of myocardial infarction and stroke by more than 80%. We recently observed that some patients have “resistant atherosclerosis”, requiring very low levels of LDL-C to stop progression, and there was no correlation between LDL-C and the progression or regression of carotid plaque burden ^79^ ( Figure 5). Both age and renal impairment increased the resistance of atherosclerosis to LDL-lowering therapy. The effect of age may in part be due to impaired renal function; by the age of 80, the average estimated glomerular filtration rate is <60 mL/minute/1.73 m ^2^ ^61^. Uremic toxins that would aggravate atherosclerosis include tHcy, ADMA, thiocyanate, and products of the intestinal microbiome, including trimethylamine n-oxide (TMAO, produced from carnitine in red meat and phosphatidylcholine in egg yolk ^80– 82^), *p*-cresyl sulfate, and other toxins produced from amino acids ^61^. I now have over 100 patients with LDL-C <0.5 mmol/L (19 mg/dL) and a handful with LDL-C <0.3 mmol/L (12 mg/dL) because that’s what it took to stop plaque progression.

There is no reason to be concerned about low levels of LDL-C. Adverse effects of statins are not due to low levels of LDL-C, and many of the putative adverse effects of statins are myths: statins probably do not cause hepatotoxicity, renal impairment, intracerebral hemorrhage ^83– 85^, or cognitive impairment ^73^. In the pooled analysis of trials with alirocumab ^86^, there were no intracerebral hemorrhages reported, even with LDL-C <15 mg/dL, and in the evolocumab trial ^75^ there was no significant difference in hemorrhagic stroke between evolocumab and placebo. Likewise, very low levels of LDL-C from therapies directed at PCSK9 do not appear to cause myopathy, diabetes, or cognitive impairment ^75, 86^.

## Conclusion

In 2007, we estimated ^87^ that about 80% of recurrent strokes could be prevented by a combination of lifestyle changes and appropriate medication. Recently, I reviewed rational medical therapy for stroke prevention ^88^. With these recent advances in hypertension, nutrition, anticoagulation, antiplatelet therapy, intracranial stenosis, and lipid-lowering therapy, it is likely that recurrent stroke could be reduced even more. The key to preventing recurrent stroke is to identify the cause of the initial event so that therapy can be individualized to the patient. While the vascular risk factors should be treated in all patients, anticoagulation and PFO closure would be appropriate in only some patients.
